# Vulvar malignant melanoma versus vaginal malignant melanoma: distinct biology, distinct clinical behavior, and distinct management?

**DOI:** 10.3389/fonc.2026.1907495

**Published:** 2026-07-23

**Authors:** Fangyu Xu

**Affiliations:** Department of Gynecology, Gynecology Department, Hubei Provincial Hospital of Integrated Chinese and Western Medicine, Wuhan, China

**Keywords:** female genital tract melanoma, mucosal melanoma, vaginal malignant melanoma, vulvar malignant melanoma, vulvovaginal malignant melanoma

## Abstract

Malignant melanoma of the female genital tract is rare but highly aggressive. Vulvar malignant melanomas and vaginal malignant melanomas are commonly grouped as vulvovaginal malignant melanoma because of their anatomical proximity, low incidence, and limited disease-specific evidence. However, accumulating clinical, pathological, and biological observations suggest that this conventional grouping may mask important site-related heterogeneity. Vulvar malignant melanoma arises within a complex mucocutaneous transition zone and may partially overlap biologically with cutaneous melanoma, whereas vaginal malignant melanoma more consistently demonstrates features of mucosal melanoma. These distinctions influence patterns of presentation, local extension, lymphatic dissemination, therapeutic feasibility, recurrence behavior, and survival outcomes. Vaginal malignant melanoma is frequently diagnosed at a more advanced stage because of occult symptoms and challenging local control, contributing to its particularly poor prognosis. In contrast, vulvar malignant melanoma encompasses a broader biological spectrum that may require subsite-specific interpretation. Although both entities belong to the spectrum of lower female genital tract melanoma, emerging evidence indicates that they should not be treated as interchangeable diseases in either research design or clinical management. This review synthesizes current evidence regarding the overlap and divergence between vulvar and vaginal malignant melanoma and advocates for a site-a^1^ware framework to guide future translational research, surgical decision-making, and systemic treatment strategies.

## 1 Introduction

Malignant melanoma of the female genital tract is a rare but highly aggressive malignancy (1). Vulvar malignant melanoma and vaginal malignant melanoma are frequently grouped together as vulvovaginal malignant melanoma because of their anatomical proximity, low incidence, and the limited availability of site-specific evidence derived mainly from retrospective series and database studies (2). Although this classification has practical value for data aggregation, it may also imply that these tumors represent a single biological and clinical entity (3). Increasing evidence suggests that this assumption may be overly simplistic and could obscure clinically meaningful differences relevant to diagnosis, treatment, and prognosis.

The rationale for routinely combining vulvar and vaginal malignant melanoma warrants re-evaluation. Despite sharing features of rare non-ultraviolet-associated melanocytic malignancies, the two tumors differ in anatomical context, surgical accessibility, lymphatic drainage, recurrence patterns, and survival outcomes (4). Emerging molecular data further support this distinction. Vaginal malignant melanoma appears to align more closely with conventional mucosal melanoma, characterized by lower tumor mutational burden, frequent *KIT* alterations, and relatively poor responses to immune checkpoint inhibitors (5). In contrast, vulvar malignant melanoma demonstrates greater molecular heterogeneity, with some tumors showing features overlapping with cutaneous melanoma, including occasional *BRAF* mutations and ultraviolet-signature changes (6). Given its location across the cutaneous–mucosal transition zone, vulvar malignant melanoma may represent a biologically transitional category rather than a uniformly mucosal subtype (7).

**Figure 1:**
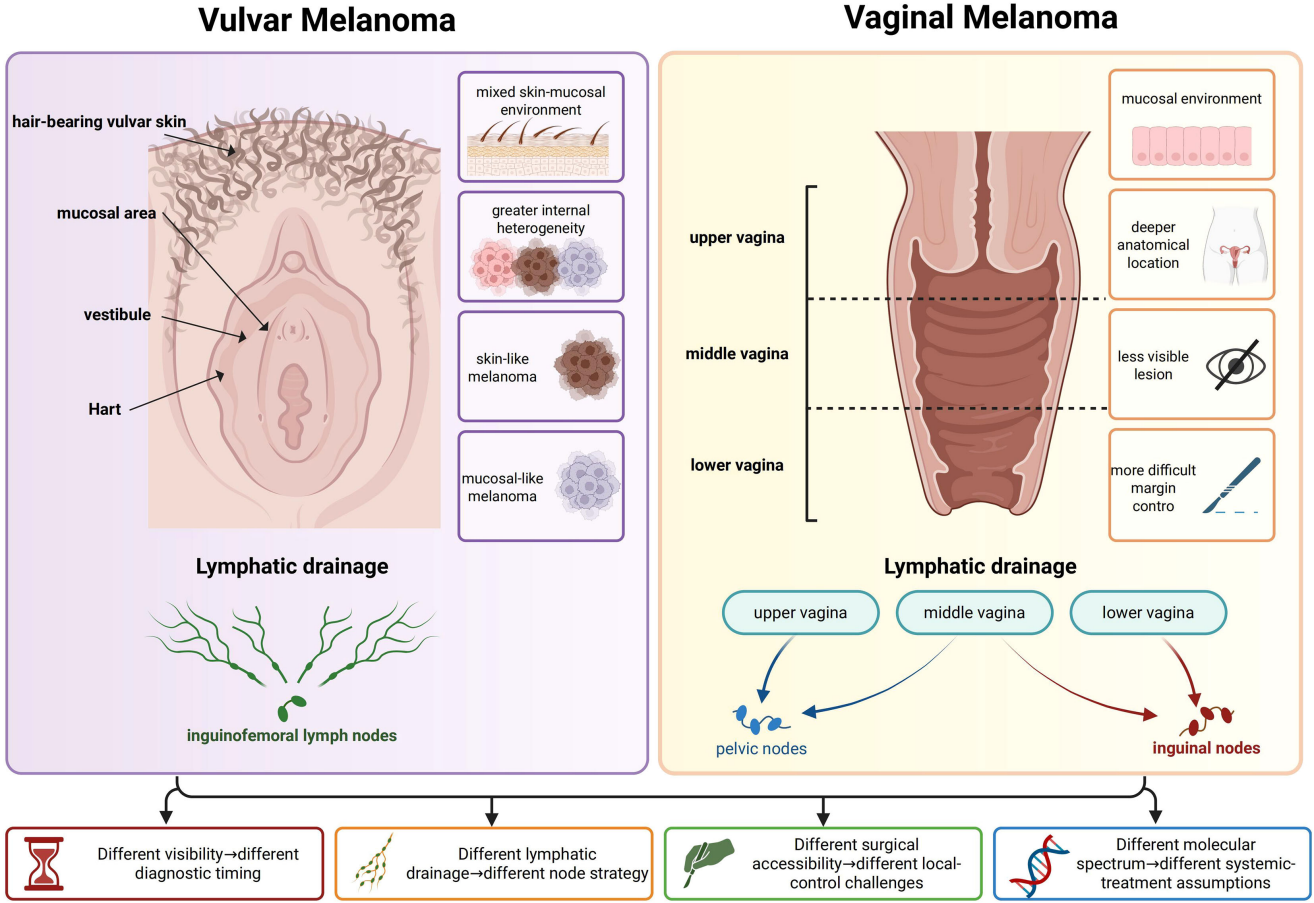
Schematic illustration of anatomical and biological differences between vulvar and vaginal malignant melanoma. Created in BioRender. Gao, Y. (2026) https://BioRender.com/cjek853.

This review critically examines whether the current umbrella term “vulvovaginal malignant melanoma” remains clinically appropriate. By comparing vulvar and vaginal malignant melanoma across anatomical, molecular, pathological, staging, surgical, systemic-treatment, and prognostic domains (**Table 1**; **Figure 1**), we aim to clarify whether a more site-specific framework is needed to guide future research and individualized clinical management.

**Table 1:** Site-specific differences between vulvar malignant melanoma and vaginal malignant melanoma.

Domain	Vulvar malignant melanoma	Vaginal malignant melanoma	Clinical implication
Anatomy/biology	Cutaneous-mucosal junction; heterogeneous phenotype; mixed biology (50, 51)	Pure mucosal origin; highly invasive (50)	More consistent aggressive biology
Detection	Externally visible; earlier detection; shorter delay (7, 50)	Intracavitary lesion; occult onset; delayed diagnosis (52)	Frequent advanced-stage presentation
Clinical signs	Pigmented plaque/mass; ulceration; pruritus/bleeding (50)	Abnormal bleeding; pelvic discomfort (52)	High risk of gynecologic misdiagnosis
Pathology	Diverse subtypes; Breslow-based assessment; clearer margins (50)	Nodular invasion; difficult staging; uncertain margins (50)	Higher staging and margin uncertainty
Molecular profile	*KIT*-enriched *(*5); occasional *BRAF*	*NRAS*-enriched; low *KIT*/*BRAF (*5); fewer targets	Routine *BRAF/KIT/NRAS* testing
Staging	AJCC melanoma TNM; relatively standardized; skin-type pathway (12)	No dedicated system; FIGO limitations (50)	Separate reporting recommended
Surgery	Accessible margins; organ-sparing options; lower morbidity (32)	Deep location; adjacent organs; wider resection (52)	Greater functional-surgical burden
Complications/QoL	Wound issues; scarring; lymphedema (4)	Vaginal stenosis; urinary-bowel injury; sexual dysfunction (4)	Greater long-term QoL impairment
Lymphatic spread	Inguinal drainage; feasible SLNB (12, 50)	Pelvic and inguinal drainage; unreliable SLNB (12, 52); complex mapping	Nodal assessment less reliable
Treatment timing	Adjuvant therapy; salvage therapy; recurrence setting (28, 32)	Neoadjuvant/conversion therapy; early systemic therapy; palliation (28, 32)	Greater systemic-treatment dependence
Progression pattern	Locoregional recurrence; late distant spread (32, 53)	Early hematogenous spread; pelvic relapse; distant metastasis (52)	Earlier systemic progression
Overall prognosis	SEER-18 reported median disease-specific survival of 99 months for vulvar melanoma (95% CI, 60-138) (50)	SEER-18 reported median disease-specific survival of 19 months for vaginal melanoma (95% CI, 16-22) (50)	Primary site as prognostic factor

*KIT*, proto-oncogene, receptor tyrosine kinase; *NRAS*, Neuroblastoma RAS viral oncogene homolog; *BRAF*, B-Raf proto-oncogene; SLNB, sentinel lymph-node biopsy; QoL, quality of life.

## 2 Methods

### 2.1 Review design

This study was designed as a narrative review comparing vulvar malignant melanoma and vaginal malignant melanoma across anatomical, clinicopathological, molecular, staging, therapeutic, prognostic, and survivorship-related domains. The aim was to synthesize available evidence and evaluate whether these tumors should be interpreted within a site-specific framework rather than as a single undifferentiated vulvovaginal malignant melanoma category.

### 2.2 Search strategy and information sources

A literature search was conducted in PubMed/MEDLINE, Embase, Web of Science, Scopus, and the Cochrane Library from database inception to May 2026. Search terms included “vulvar malignant melanoma”, “vulval melanoma”, “vaginal malignant melanoma”, “female genital tract melanoma”, “mucosal melanoma”, “*KIT*”, “*BRAF*”, “*NRAS*”, “staging”, “surgery”, “sentinel lymph node biopsy”, “immunotherapy”, “targeted therapy”, “recurrence”, “prognosis”, and “quality of life”. Relevant guidelines, consensus statements, and reference lists were also reviewed.

### 2.3 Eligibility criteria and study selection

Eligible publications included cohort studies, population-based studies, institutional case series, molecular profiling studies, reviews, meta-analyses, clinical guidelines, and consensus documents reporting data on vulvar malignant melanoma, vaginal malignant melanoma, or vulvovaginal malignant melanoma. Studies were prioritized when they provided site-specific information on pathology, molecular alterations, staging, treatment, recurrence, survival, or survivorship. Non-melanoma tumors, benign melanocytic lesions, unrelated cutaneous melanoma studies, and duplicate reports without additional relevant data were excluded.

### 2.4 Data extraction, synthesis, and evidence appraisal

No formal risk-of-bias scoring tool was applied because of the narrative design; however, evidence was interpreted according to study design, sample size, anatomical specificity, and clinical relevance. Data were extracted on tumor site, pathology, molecular features, staging, surgery, nodal assessment, systemic therapy, recurrence, prognosis, and quality of life. Because available evidence was mostly retrospective and heterogeneous, findings were synthesized narratively. Greater weight was given to guidelines, consensus statements, systematic reviews, population-based studies, multicenter series, and studies with site-specific data.

## 3 Why have vulvar malignant melanoma and vaginal malignant melanoma commonly been grouped together?

Traditionally, vulvar malignant melanoma and vaginal malignant melanoma have been discussed as a single category. This approach has several reasons (7). First, both tumors arise in the lower female genital tract and represent rare melanocytic malignancies; because of their rarity, individual institutions have difficulty accumulating sufficiently large case series. Second, compared with conventional cutaneous melanoma, melanomas arising in the vulvovaginal region are generally included in the spectrum of rare-site mucosal melanomas, which are characterized by more aggressive biological behavior and poorer prognosis. This unified classification has encouraged clinicians and researchers to regard vulvar and vaginal malignant melanoma as related disease entities, particularly when discussing the biology of rare-site melanoma, the scarcity of evidence for systemic therapy, and the need to extrapolate from broader clinical experience in mucosal melanoma. Third, both diseases are associated with high recurrence rates, limited prospective evidence, and outcomes inferior to those of common cutaneous melanoma (8). Both raise similar questions regarding the role of conservative surgery, the value of nodal assessment, the contribution of radiotherapy to local control, and the uncertain efficacy of systemic therapy in rare-site disease. These shared challenges have provided the basis for their combined discussion in previous literature (7, 8).

For these reasons, grouping vulvar and vaginal malignant melanoma together is convenient. Nevertheless, convenience in data organization and evidence synthesis should not be mistaken for biological or clinical equivalence (9). With increasing attention to anatomical context, molecular variation, surgical constraints, and prognostic patterns, a growing body of evidence suggests that vulvar and vaginal malignant melanoma should no longer be managed or interpreted as a single undifferentiated entity (10).

## 4 Differences between vulvar malignant melanoma and vaginal malignant melanoma

### 4.1 Anatomical differences

The vulva includes areas that are clearly cutaneous as well as areas adjacent to the vestibule or mucosal transition zone. Therefore, not all vulvar malignant melanomas are biologically identical: some lesions more closely resemble cutaneous melanoma clinicopathologically, whereas others are more consistent with mucosal melanoma (11). According to NCCN guidance based on lesion location, vulvar malignant melanoma located outside Hart’s line of the vulvar vestibule is defined as cutaneous-type disease, whereas lesions located inside Hart’s line are defined as mucosal-type disease (12). In contrast, vaginal malignant melanoma predominantly arises in a purely mucosal environment. Grouping the two entities together may therefore obscure important internal heterogeneity and reduce the accuracy of comparisons between vulvar and vaginal lesions.

### 4.2 Clinical presentation and diagnostic course

Clinical presentation and diagnostic pathways constitute an important distinction between vulvar malignant melanoma and vaginal malignant melanoma. Although both are rare melanocytic malignancies of the lower female genital tract, their symptom patterns, lesion detectability, and timing of diagnosis differ substantially because of anatomic location (13).

Vulvar malignant melanoma usually presents as a pigmented macule, plaque, or nodular lesion involving the labia majora, labia minora, clitoris, or adjacent vulvar structures. Common symptoms include pruritus, irritation, bleeding, ulceration, or changes in a pre-existing pigmented lesion. Because the vulva is externally visible and readily accessible for examination, lesions are more likely to be detected earlier by patients or clinicians, facilitating prompt biopsy and histopathologic confirmation. In large retrospective series, approximately 70–75% (4) of vulvar malignant melanoma cases are diagnosed as localized disease, although regional nodal involvement is still reported in nearly 20–30% (14) of patients at presentation. Median Breslow thickness commonly exceeds 3–4 mm, indicating that delayed recognition remains clinically relevant despite easier visibility (14).

In contrast, vaginal malignant melanoma often follows a more occult clinical course. The vagina is an internal organ, and early lesions may remain asymptomatic until substantial local growth occurs. The most frequent presenting manifestations are postmenopausal bleeding, irregular vaginal bleeding, abnormal discharge, pelvic discomfort, or detection of an intravaginal mass. These symptoms are nonspecific and frequently overlap with benign gynecologic disorders, contributing to diagnostic delay (7). Retrospective studies of vaginal malignant melanoma have reported median tumor sizes of approximately 3 cm and median intervals of about 3 months between symptom onset and diagnosis, although substantially longer delays have also been described. Furthermore, amelanotic or weakly pigmented lesions are relatively common and may complicate clinical recognition and biopsy selection (15).

These differences in lesion detectability appear clinically meaningful. Compared with vulvar malignant melanoma, vaginal malignant melanoma is more frequently diagnosed with deeply invasive tumors, advanced local extension, or nodal involvement at presentation. Several retrospective series have demonstrated poorer local control and inferior survival outcomes in vaginal malignant melanoma, likely reflecting delayed diagnosis, greater tumor thickness, and the technical challenges of achieving adequate surgical margins within the vaginal anatomy.

### 4.3 Pathology

Histopathologically, vulvar and vaginal malignant melanomas may show mucosal lentiginous, nodular, and mixed growth patterns, but their distribution and diagnostic implications differ. Recent vulvar malignant melanoma series emphasize systematic reporting of ulceration, mitotic activity, necrosis, margin status, and other prognostically relevant features (7) (**Table 2**). Vulvar malignant melanoma may overlap morphologically with cutaneous melanoma, particularly when hair-bearing skin or the mucocutaneous junction is involved, whereas vaginal malignant melanoma more consistently reflects mucosal melanoma biology. Vaginal malignant melanoma often presents as an aggressive vertically growing tumor with occult submucosal extension and difficult margin assessment. Current NCCN and melanoma-related guidelines recommend standardized evaluation of tumor thickness, ulceration, margins, nodal status, and clinically actionable risk factors (16). However, Breslow thickness is generally more reproducible in vulvar malignant melanoma than in vaginal malignant melanoma, where thin mucosa, fragmentation, and specimen orientation may compromise measurement (7). Preferentially Expressed Antigen in Melanoma immunohistochemistry can help distinguish melanoma from benign melanocytic proliferations and may aid assessment of subtle margin involvement (17).

**Table 2:** Comparative pathology and molecular features of vulvar and vaginal malignant melanoma.

Feature	Vulvar malignant melanoma	Vaginal malignant melanoma	Clinical implication
Histologic subtype	Lentiginous/mucosal type; nodular type; superficial spreading (50)	Predominantly nodular; invasive mass; limited *in situ* phase (50)	Vulvar precursor phase; vaginal *de novo* invasion
*BRAF* mutation	Low frequency; test routinely; non-V600 variants (50, 51)	Low frequency; rare V600; noncanonical variants (50, 51)	Limited *BRAF*-targeted eligibility
*KIT* mutation	Relatively common; 21.6%-27% (50, 51)	Lower frequency; approximately 8% (50);	Key actionable target in vulvar malignant melanoma
*NRAS* mutation	Lower frequency; approximately 10.2% (50); variable association	Higher frequency; nodular lesions (50); MAPK activation	Greater *MAPK*-pathway dependence

From a molecular-genetic perspective, major guidelines (12) commonly recommend testing for *BRAF*, *KIT*, and *NRAS* mutations, but the distribution of these alterations may differ between the two diseases. *BRAF* mutations are frequently observed in cutaneous melanoma and have been reported to reach approximately 40%–60% (12, 18), but appear to be uncommon in mucosal melanoma, often reported at less than 5% (19); in addition, available retrospective data suggest that mucosal-type vulvar malignant melanoma may have a slightly higher *BRAF* mutation rate than vaginal mucosal melanoma (20), although this observation requires further validation. Molecularly, the targeted-treatment rationale for vulvar and vaginal malignant melanoma may not be identical. ESMO, NCCN, and other guidelines emphasize that advanced or high-risk melanoma should undergo testing for actionable alterations. *BRAF* V600 is generally considered a key target, and *NRAS* and *KIT* may also be included in molecular classification to guide targeted therapy or clinical-trial enrollment. *BRAF* mutations are common in cutaneous melanoma and have been reported in approximately 37%–60% of cases (18), whereas BRAF alterations appear to be less frequent in mucosal/vulvovaginal malignant melanoma and may more often involve non-*V600* mutations; therefore, the population eligible for *BRAF/MEK* inhibitors is likely limited in both diseases. However, because the vulvar region may include hair-bearing skin and mucocutaneous junctional components, routine *BRAF* testing should still be considered. Potential differences may involve *KIT* and *NRAS*: recent reviews and retrospective series suggest that *KIT* mutations may be more frequently reported in vulvar malignant melanoma, with reported rates of approximately 22%–31% (19), than in vaginal malignant melanoma, where rates have been reported at approximately 8% (20). Therefore, *KIT* testing may be important in vulvar malignant melanoma, and patients harboring *KIT* exon 11 or exon 13 mutations may benefit from *KIT* inhibitors such as imatinib. Conversely, *NRAS* mutations appear relatively more common in vaginal malignant melanoma. At present, *NRAS* lacks a mature direct targeted therapy, but it may indicate *MAPK*-pathway dependence and clinical-trial relevance (21). Overall, available evidence suggests that targeted-treatment opportunities in vulvar malignant melanoma may be more oriented toward screening for actionable *KIT* and *BRAF* alterations, whereas vaginal malignant melanoma may be associated with relatively more *NRAS*-related biology and a potentially lower probability of benefit from *BRAF*- or *KIT*-directed therapy; however, these site-related molecular differences require further validation in larger studies (22, 23).

It should be noted that both vulvar malignant melanoma and vaginal malignant melanoma are rare malignancies with substantial molecular heterogeneity, and current evidence does not support a simplified inference of biological behavior or molecular profile based solely on anatomical site (24, 25). Vulvar malignant melanoma itself should not be regarded as a single biological entity, as it may arise from keratinized or hair-bearing vulvar skin, the mucocutaneous junction, or vulvar mucosal surfaces. Therefore, when discussing clinicopathological features and molecular testing strategies, it is important to distinguish, whenever possible, among cutaneous-type vulvar malignant melanoma, mucosal-type vulvar malignant melanoma, and primary vaginal malignant melanoma (19). Cutaneous-type vulvar malignant melanoma may be assessed with reference to principles applied to cutaneous melanoma, particularly with respect to certain actionable alterations such as *BRAF* mutations. However, it would be inappropriate to generalize all vulvar malignant melanomas as being biologically similar to cutaneous melanoma. In contrast, melanomas arising from vulvar mucosal sites or the mucocutaneous junction should be interpreted in the context of the biological characteristics of mucosal melanoma (20, 23).

### 4.4 Staging systems 

Staging for vulvar and vaginal malignant melanoma should be presented as a hierarchy of reporting frameworks rather than as a single universally validated rule (**Table 3**). The NCCN Vulvar Cancer guideline provides the most explicit site-based guidance: it distinguishes cutaneous vulvar malignant melanoma from mucosal vulvovaginal malignant melanoma using Hart’s line and recommends clinical staging with the AJCC melanoma TNM framework, while linking biopsy, pathology, imaging, margin assessment, sentinel-node evaluation, systemic therapy, and *BRAF/KIT* testing to melanoma-specific management pathways (26). UICC TNM 9th edition provides the internationally accepted anatomic TNM language, but recent ano-uro-genital mucosal melanoma guidance emphasizes that skin-melanoma TNM may be appropriate for smaller tumors involving vulvar skin and may be less anatomically precise for labia minora, vestibular, vaginal, urethral, or other mucosal sites (27) CSCO 2025 and CACA 2025 are therefore retained as current Chinese melanoma diagnosis-and-treatment guidelines, especially for domestic management context, but they are not presented as independent vulvovaginal-melanoma-specific staging systems (27).

**Table 3:** Staging frameworks for vulvar and vaginal malignant melanoma.

Authority/year	Vulvar malignant melanoma staging	Vaginal malignant melanoma staging	Key reporting message
NCCN Vulvar Cancer v3.2024 (26)	AJCC melanoma TNM; Hart-line subclassification;	AJCC melanoma TNM suggested; mucosal pathway;	Pragmatic AJCC-based staging; institutional variability
UICC TNM 9th/RCPath 2025 (54)	Cutaneous melanoma TNM; vulva included; Breslow/ulceration/nodes/LDH	No skin TNM; Descriptive staging	Discordance with NCCN
*FIGO* vulvar cancer 2025 (27)	Vulvar-cancer FIGO; melanoma excluded; not recommended	Not applicable;	Avoid FIGO vulvar-cancer staging
FIGO vaginal cancer 2025 (27)	Not applicable	Epithelial-tumor focus; melanoma excluded; mucosal melanoma entity	Avoid FIGO vaginal-cancer staging
CSCO 2025 framework (55)	Cutaneous-type vulva included; AJCC/pTNM; Breslow-based	No pTNM system; mucosal framework; clinical description	China framework: vulva stageable, vagina not
CACA 2025 framework (56)	Hart-line classification; unified AJCC staging; subtype-adapted management	Mucosal melanoma; no standard staging; descriptive assessment	Clearest domestic distinction

For vulvar malignant melanoma, staging should first distinguish cutaneous-type from mucosal-type disease. When a tumor arises from hair-bearing vulvar skin or has a clearly cutaneous origin, AJCC/TNM cutaneous melanoma variables remain the most interpretable reporting framework. Breslow thickness, ulceration, margin status, nodal assessment, and distant metastasis should be explicitly recorded. For tumors arising from the vestibule or mucosal vulva, these melanoma-related variables should also be reported, but the manuscript should avoid implying that prognostic evidence from cutaneous melanoma is directly transferable. FIGO vulvar-carcinoma staging may describe local anatomic extension, but it should not be cited as a melanoma-specific staging system (20, 28).

For primary vaginal malignant melanoma, the staging limitation is greater. Current evidence indicates that female lower genital tract melanoma lacks a unified, prospectively validated staging system, and clinical practice varies across anatomic site, specialty, and institution (29). FIGO vaginal-cancer staging offers a familiar gynecologic anatomic framework and emphasizes clinical examination and imaging for local extent. However, it was designed for vaginal carcinoma rather than melanoma biology and does not adequately capture Breslow thickness, ulceration, early hematogenous dissemination, molecular subtype, or immune response. Conversely, AJCC cutaneous melanoma staging captures melanoma-specific prognostic factors but does not fully represent vaginal level, paravaginal extension, cervicovaginal involvement, or pelvic-organ invasion.

Therefore, vulvar malignant melanoma reports should include tumor location relative to Hart’s line, AJCC/TNM melanoma variables, Breslow thickness, ulceration, margin status, nodal basin, and distant disease. Vaginal malignant melanoma reports should include tumor level, local extension, measurable melanoma parameters, nodal and distant status, and FIGO descriptors only as non-melanoma-specific anatomic information. Thus, vaginal malignant melanoma does not lack staging terminology; rather, it lacks a validated melanoma-specific staging framework.

### 4.5 Surgical management

Surgical recommendations require caution because no operative strategy for vulvar or vaginal malignant melanoma is supported by robust prospective, site-specific evidence (3, 30). Most available data come from retrospective institutional cohorts, registry analyses, and small case series with heterogeneous disease stages, treatment eras, margin definitions, surgical techniques, and adjuvant therapies (4). Vulvar and vaginal malignant melanomas are rare lower genital tract tumors, but their surgical management differs because of anatomical location, operative complexity, and functional consequences. For both diseases, the general goal is complete excision with microscopically negative margins when feasible, while minimizing functional impairment (31). Treatment should therefore rely on multidisciplinary assessment and individualized balancing of local control, quality of life, surgical morbidity, and systemic recurrence risk. Consequently, surgery should be regarded primarily as a local-control strategy rather than as a proven intervention for eliminating systemic relapse risk (32).

For vulvar malignant melanoma, wide local excision or partial vulvectomy may be considered when complete resection with acceptable morbidity is feasible (3, 30). Surgery aims to obtain negative margins while preserving the clitoris, urethral meatus, anus, perineal body, and vulvar morphology and function. Because many vulvar lesions are externally visible, preoperative mapping and margin assessment may be relatively straightforward compared with vaginal malignant melanoma. For melanomas involving the hair-bearing skin of the vulva, excision margins may be guided by Breslow thickness-based principles established for cutaneous melanoma, while taking into account site-specific anatomical and functional limitations (3). However, the applicability of these principles to vulvar malignant melanoma has not been confirmed in prospective studies. Routine total vulvectomy or destructive procedures should generally be avoided when adequate margins can be achieved with less radical surgery (33). Primary clitoral melanoma further illustrates the internal heterogeneity of vulvar malignant melanoma. Because the clitoris has substantial sensory and sexual functional importance, surgical planning should not be based solely on the pursuit of wider anatomical resection. A recent site-specific systematic review suggested that organ-preserving wide local excision should be preferred when microscopically negative margins can be achieved, although this recommendation is based on only 15 reported cases and requires cautious interpretation (34).

Given the rarity of vaginal malignant melanoma, no surgical approach is supported by robust prospective comparative evidence. Surgery should be individualized according to tumor site, size, depth of invasion, focality, exposure, suspected submucosal extension, and adjacent-organ involvement. Superficial and well-exposed lesions may be managed with wide local excision or partial vaginectomy, whereas bulky, deeply invasive, upper vaginal, or cervicovaginal tumors may require partial or total vaginectomy (32). Radical or en bloc resection should generally be reserved for cases with suspected or confirmed adjacent-organ invasion, or when such resection is necessary to achieve negative margins (35). Routine prophylactic removal of clinically uninvolved pelvic organs is not supported by current evidence and is not routinely recommended (35). Current evidence does not support routine removal of the uterus, cervix, bladder, rectum, or other clinically uninvolved pelvic organs solely to increase surgical radicality in the absence of direct invasion, because such procedures may substantially increase morbidity without proven survival benefit (35).

Technically, vulvar surgery requires balancing the need for negative margins with preservation of urinary, sexual, and anal function. Vaginal surgery is often more challenging because the tumor lies within a narrow canal, complicating exposure, localization, assessment of submucosal extension, and deep margin control. Postoperative morbidity also differs: vulvar surgery mainly affects wound healing, vulvar function, and the inguinal nodal basin, whereas vaginal surgery more often compromises urinary, rectal, pelvic-floor, and sexual function and may cause vaginal stenosis or canal loss. Thus, both diseases require avoidance of excessive surgery without proven survival benefit, especially vaginal malignant melanoma, which demands individualized multidisciplinary decision-making (32).

### 4.6 Lymphatic drainage and intraoperative nodal management

The anatomical feasibility, staging value, prognostic value, and therapeutic benefit of sentinel lymph-node biopsy (SLNB) should be considered separately in vulvovaginal malignant melanoma. In vulvar malignant melanoma, lymphatic drainage is generally more predictable than in vaginal malignant melanoma, with the superficial and deep inguinofemoral nodes representing the main regional basins; however, drainage may be bilateral, particularly for midline, clitoral, or vestibular tumors. This relatively accessible pattern makes lymphatic mapping and SLNB technically feasible in selected clinically node-negative patients.Available evidence, including multicenter experience and systematic review data, suggests that SLNB can detect occult nodal metastases in a subset of patients with vulvar malignant melanoma, but the evidence remains limited by the rarity of the disease, small sample sizes, retrospective design, and heterogeneity in tumor characteristics, surgical technique, and adjuvant treatment (14, 36). Primary clitoral melanoma represents an exceptionally rare vulvar subsite. Owing to the midline location of the clitoris and the possibility of drainage to both inguinal basins, unilateral nodal evaluation may result in incomplete staging. The available clitoral-melanoma review therefore supports consideration of bilateral lymphatic mapping and bilateral SLNB in clinically node-negative patients when technically feasible and when the result is expected to influence subsequent management (34).

The main value of SLNB in vulvar malignant melanoma is therefore staging and risk stratification rather than established therapy. A negative SLNB may refine nodal staging and help avoid unnecessary inguinofemoral lymphadenectomy in selected patients, whereas a positive SLNB confirms regional nodal involvement and may inform prognosis, adjuvant systemic therapy discussions, radiation-field planning in selected cases, clinical-trial eligibility, and surveillance intensity. Nevertheless, these should be interpreted as staging and prognostic benefits. A survival benefit from SLNB itself has not been demonstrated in vulvar malignant melanoma. Retrospective associations between nodal assessment and improved overall survival should be interpreted cautiously, because they may reflect stage migration, patient selection, differences in baseline fitness, treatment era, and access to multidisciplinary care rather than a direct therapeutic effect of SLNB. Thus, in clinically node-negative vulvar malignant melanoma, SLNB may be considered on an individualized basis after assessment of Breslow thickness, ulceration, tumor location, imaging findings, surgical morbidity, patient preference, and whether the result would alter subsequent management (36, 37).

By contrast, routine SLNB should not be implied for primary vaginal malignant melanoma. Vaginal lymphatic drainage is segmental and variable: lower vaginal tumors may drain to inguinal nodes, whereas upper vaginal tumors may drain to pelvic, obturator, internal iliac, external iliac, presacral, or para-aortic pathways, and bidirectional drainage may occur. This variability reduces the predictability and reproducibility of sentinel-node mapping. Moreover, evidence supporting SLNB in vaginal malignant melanoma is restricted mainly to case reports, small institutional experiences, and extrapolation from vulvar cancer, cervical cancer, and cutaneous melanoma; these data are insufficient to validate SLNB as a routine procedure for vaginal malignant melanoma. In clinically node-negative vaginal malignant melanoma, SLNB should therefore be reserved, if used at all, for highly selected patients treated at experienced centers, particularly for lower vaginal lesions in which preoperative mapping is feasible and the result has a clear management consequence. Otherwise, nodal evaluation should rely on careful physical examination, cross-sectional imaging, PET/CT when clinically indicated, and biopsy of suspicious nodes (36, 38, 39).

Management of clinically or pathologically positive nodes should also be individualized. Therapeutic lymphadenectomy, nodal radiotherapy, and systemic therapy may each be appropriate depending on the involved basin, nodal burden, symptoms, resectability, performance status, and distant disease assessment. Importantly, randomized evidence in cutaneous melanoma has shown that immediate completion lymph-node dissection after a positive sentinel node improves regional control but does not improve melanoma-specific survival; although this evidence cannot be directly transferred to vulvovaginal malignant melanoma, it supports caution against interpreting nodal surgery as inherently survival-prolonging. Overall, SLNB is anatomically more feasible and potentially useful for staging and prognosis in selected vulvar malignant melanoma, but its survival benefit remains unproven, and its routine usefulness in vaginal malignant melanoma is not established (37, 38).

### 4.7 Systemic therapy

The systemic management of vulvar and vaginal malignant melanoma relies primarily on immune-checkpoint inhibitors, targeted therapy, and clinical-trial enrollment; however, evidence remains limited because mucosal melanoma is markedly underrepresented in prospective melanoma trials, with vulvar and especially vaginal malignant melanoma comprising only a very small subset (3). Consequently, most therapeutic recommendations are extrapolated from cutaneous or pooled mucosal melanoma data, which may not adequately reflect the distinct biology and treatment responsiveness of vulvovaginal disease.

For unresectable or metastatic disease, anti-*PD*-1 monotherapy or anti-*PD*-1 plus anti-*CTLA*-4 combinations remain the principal systemic options (3). Retrospective database and institutional studies suggest that immunotherapy may improve survival in selected patients, although responses are heterogeneous and generally less durable than those observed in cutaneous melanoma (19). This limitation appears particularly relevant in vaginal malignant melanoma, which demonstrates biologic features associated with poor immunotherapy responsiveness, including lower tumor mutational burden, reduced immunogenicity, and highly aggressive local recurrence patterns. These characteristics likely contribute to the consistently inferior prognosis of vaginal malignant melanoma and support the need for biology-driven rather than purely stage-driven therapeutic strategies.

Systemic treatment intent also differs between the two entities. Vulvar malignant melanoma is more frequently diagnosed at a surgically resectable stage; therefore, systemic therapy is commonly incorporated as postoperative adjuvant treatment or for recurrent/metastatic disease control. In contrast, vaginal malignant melanoma is often occult and presents with locally advanced, pelvic, or metastatic disease, necessitating earlier systemic intervention in neoadjuvant, conversion, or palliative settings. Although immune-checkpoint inhibitor combinations may reduce tumor burden in selected advanced cases, prospective site-specific evidence remains scarce.

Comprehensive molecular profiling is essential in both tumors. Testing for *BRAF, KIT*, and *NRAS* alterations is recommended to guide targeted therapy or clinical-trial enrollment (3). Conventional chemotherapy is now largely reserved for salvage treatment when immunotherapy, targeted therapy, or trials are ineffective or unavailable (40). Emerging neoadjuvant anti-*PD-1* plus antiangiogenic strategies in mucosal melanoma remain promising but should be extrapolated cautiously to vulvovaginal malignant melanoma (41).

### 4.8 Recurrence patterns and prognosis

Differences in recurrence patterns, site-specific anatomy, and prognosis provide important clinical grounds for distinguishing vulvar malignant melanoma from vaginal malignant melanoma (**Table 4**). Compared with vulvar malignant melanoma, vaginal malignant melanoma generally shows poorer survival and a greater tendency toward early local or distant failure. Recent institutional and single-center surgical studies have confirmed significantly worse survival for vaginal malignant melanoma, high recurrence rates, frequent distant metastasis, and poor 5-year relative overall survival in vulvovaginal malignant melanoma. Differences in recurrence-free survival further indicate the propensity of vaginal malignant melanoma for early relapse. Recurrence may occur rapidly even after local excision or radical surgery, suggesting that conventional local-control strategies are often insufficient. These findings support early multidisciplinary evaluation, intensive postoperative surveillance, and consideration of perioperative systemic therapy or clinical trials when appropriate.

**Table 4:** Recurrence patterns and prognosis in vulvar and vaginal malignant melanoma.

Comparative domain	Vulvar malignant melanoma	Vaginal malignant melanoma
Recurrence rate	In a 2025 Chinese retrospective cohort, recurrence occurred in 6/11 vulvar cases; overall 2-year recurrence was 82% (29)	In the same cohort, recurrence occurred in 10/14 vaginal cases; another 2025 cohort reported 5-year progression of 72% overall, not site-specific (29, 55)
Median recurrence-free survival	median PFS 33 months in a 2026 vulvar/vaginal cohort (56)	median PFS 5 months in the 2026 cohort (56).
Recurrence pattern	Predominantly local recurrence (60%-70%) (32, 50)	High rates of both local and distant recurrence (distant recurrence: 70%-80%) (44).
Metastatic features	Hematogenous spread mainly occurs in advanced disease; nodal metastasis predominates (29).	Early hematogenous spread is common, particularly to the lung and liver.
Speed of recurrence	Relatively slower; within 1–2 years after surgery.	Very rapid; within an average of 10 months after surgery (44).
Difficulty of disease control	Local disease is more controllable, repeat surgery feasible (29, 32),.	Local control is difficult, distant metastases are challenging to manage (50, 53)

The recurrence patterns of vulvar malignant melanoma and vaginal malignant melanoma differ substantially. Vulvar malignant melanoma more often allows visible local assessment and anatomically defined inguinal nodal evaluation, although local, regional, and distant recurrence remain clinically relevant. In contrast, vaginal malignant melanoma carries higher risks of pelvic recurrence and distant metastasis, partly owing to occult mucosal growth, difficult margin control, and rich pelvic vascular and lymphatic networks (42). Site-specific anatomical factors may further explain these outcomes. For vulvar malignant melanoma, inguinal nodal involvement is a key prognostic variable. For vaginal malignant melanoma, tumors in surgically challenging regions, such as the upper vagina or posterior wall, may have higher risk because of deep pelvic anatomy and proximity to the bladder and rectum. Positive or close surgical margins are therefore more common, weakening local control (43). In addition, lower immunogenicity, including reduced tumor mutational burden, lower *PD-L1* positivity, and distinct immune-microenvironment features, may partly explain the poorer outcomes of vulvovaginal malignant melanoma compared with cutaneous melanoma, with vaginal malignant melanoma showing the worst prognosis (44). Recurrence should also be interpreted according to subsite.Primary clitoral melanoma represents an exceptionally rare vulvar subsite.In the published primary clitoral melanoma cases, recurrence occurred in nearly one-third of patients, and exceptionally late relapse more than 10 years after initial treatment was reported (34). Although these observations derive from a very small and heterogeneous case-based literature, they suggest that short follow-up may underestimate recurrence risk and support prolonged, subsite-aware surveillance after treatment of clitoral melanoma.

### 4.9 Quality of life and survivorship: an underrecognized but crucial dimension

Research on vulvar and vaginal malignant melanoma has focused on clinicopathological features, treatment, and survival, whereas patient-reported outcomes, sexual health, body image, psychological status, and recovery remain underexplored (**Table 5**). Given the poorer prognosis of vaginal malignant melanoma, quality-of-life outcomes should be assessed separately by primary site rather than pooled (30). Symptom burden differs by anatomy and clinical course. Vulvar malignant melanoma, owing to its superficial location, often presents with visible or palpable changes, including mass, pigmentation alteration, pruritus, ulceration, pain, or bleeding, enabling earlier recognition and symptoms largely confined to the vulva. By contrast, vaginal malignant melanoma is often occult early; advanced disease may cause bleeding, discharge, malodor, dyspareunia, pelvic pressure, and urinary or bowel obstruction, indicating broader pelvic dysfunction (7). Sexual function and body image are affected differently. In vulvar malignant melanoma, the vagina is preserved, but excision, scarring, sensory loss, and altered appearance may impair sexual comfort, femininity, and intimacy (45). In vaginal malignant melanoma, vaginectomy, vaginal shortening or occlusion, pelvic radiotherapy, and fibrosis may cause reduced lubrication, dyspareunia, penetration difficulty, or loss of penetrative capacity, with concerns about bodily integrity and self-acceptance (46, 47). Psychosocial distress in vulvar malignant melanoma may center on recurrence, appearance, and sexual confidence, whereas vaginal malignant melanoma may involve death anxiety, depression, and social withdrawal because of delayed diagnosis, aggressive treatment, poorer prognosis, and pelvic dysfunction (30, 48). Long-term limitations may be milder after limited vulvar excision but more substantial after vaginal or pelvic treatment. Future studies should include measures of quality of life, sexual function, pain, psychological distress, body image, and functional recovery alongside survival endpoints (30, 45).

**Table 5:** Disease-related quality-of-life profile in vulvar and vaginal malignant melanoma.

Domain	Vulvar malignant melanoma	Vaginal malignant melanoma
Physical symptoms	Localized symptoms; visible lesion; pruritus/bleeding (50, 51)	Occult onset; bleeding/discharge; pelvic pressure (32)
Sexual/body image	External scarring; preserved vagina; mild dyspareunia (45)	Vaginal stenosis/shortening; dryness/dyspareunia; body-integrity distress (57)
Psychosocial impact	Recurrence anxiety; appearance concerns; moderate distress (45)	Poor prognosis; treatment trauma; depression/social withdrawal (57)
Daily function	Mobility preserved; self-care maintained; limited disability (43, 52)	Pelvic pain; urinary-bowel dysfunction; impaired self-care (52, 57)

## 5 Conclusion

After comprehensive evaluation of the available evidence, vulvar and vaginal malignant melanoma should no longer be regarded as a single clinicopathological entity, but rather as anatomically related diseases with distinct biological and therapeutic characteristics. Historically, their consolidation under the umbrella term “vulvovaginal malignant melanoma” was understandable because of extreme rarity, limited datasets, and the traditional concept of mucosal melanoma as a unified category. However, this framework is increasingly inadequate in the era of precision oncology. Emerging evidence demonstrates substantial heterogeneity in molecular alterations, clinical behavior, surgical feasibility, therapeutic response, and survivorship burden between anatomical subsites. Continued indiscriminate pooling may therefore obscure clinically meaningful differences and hinder individualized management. Future research should adopt a more refined, site-specific framework by stratifying analyses according to anatomical origin, systematically reporting genomic and mutational profiles, and distinguishing cutaneous-type from mucosal-type vulvar malignant melanoma. Such reclassification is not merely semantic, but essential for improving biological understanding, optimizing treatment personalization, and advancing evidence-based care for these rare and aggressive malignancies (49).
